# Evaluation of two treatment strategies for the prevention of preterm birth in women identified as at risk by ultrasound (PESAPRO Trial): study protocol for a randomized controlled trial

**DOI:** 10.1186/s13063-015-0964-y

**Published:** 2015-09-25

**Authors:** Lourdes Cabrera-García, Sara Cruz-Melguizo, Belén Ruiz-Antorán, Ferrán Torres, Ana Velasco, Cristina Martínez-Payo, Cristina Avendaño-Solá, on behalf of the PESAPRO trial Group

**Affiliations:** Clinical Pharmacology Service, University Hospital Puerta de Hierro - Majadahonda, Manuel de Falla, 1, 28222 Majadahonda, Madrid Spain; Obstetrics and Gynecology Department, University Hospital Puerta de Hierro - Majadahonda, Manuel de Falla, 1, 28222 Majadahonda, Madrid Spain; Biostatistics and Data Management Core Facility, IDIBAPS - Hospital Clínic, 183, Mallorca Street, floor −1, 08036 Barcelona, Spain; Spanish Clinical Research Network, SCReN-IIS Puerta de Hierro Majadahonda, University Hospital Puerta de Hierro Majadahonda, Manuel de Falla, 1, 28222 Majadahonda, Madrid Spain

**Keywords:** premature birth, short cervix, vaginal progesterone, cervical pessary, prevention

## Abstract

**Background:**

Premature birth is considered one of the main problems in modern Obstetrics. It causes more than 50 % of neonatal mortality; it is responsible for a large proportion of infant morbidity and incurs very high economic costs. Cervical length, which can be accurately measured by ultrasound, has an inverse relationship with the risk of preterm birth. As a result, having an effective intervention for asymptomatic patients with short cervix could reduce the prematurity. Although recently published data demonstrates the effectiveness of vaginal progesterone and cervical pessary, these treatments have never been compared to one another.

**Methods/Design:**

The PESAPRO study is a noncommercial, multicenter, open-label, randomized clinical trial (RCT) in pregnant women with a short cervix as identified by transvaginal ultrasonography at 19 to 22 weeks of gestation. Patients are randomized (1:1) to either daily vaginal progesterone or cervical pessary until the 37th week of gestation or delivery; whichever comes first. During the trial, women visit every 4 weeks for routine questions and tests. The primary outcome is the proportion of spontaneous preterm deliveries before 34 weeks of gestation. A sample size of 254 pregnant women will be included at 29 participating hospitals in order to demonstrate noninferiority of placing a pessary versus vaginal progesterone. The first patient was randomized in August 2012, and recruitment of study subjects will continue until the end of December 2015.

**Discussion:**

This trial assesses the comparative efficacy and safety between two accepted treatments, cervical pessary versus vaginal progesterone, and it will provide evidence in order to establish clinical recommendations.

**Trial registration:**

EU Clinical Trials Register EudraCT2012-000241-13 (Date of registration: 16 January 2012); ClinicalTrials.gov Identifier NCT01643980 (Date of registration: 12 June 2012).

## Background

Preterm birth, defined as birth before 37 weeks of gestation, is the second most frequent direct cause of infant death in children younger than 5 years old [1–4]. Preterm birth is considered a major problem in modern Obstetrics, and it is the leading cause of perinatal morbidity and mortality in developed countries [5]. It is also associated with an important economic burden [6, 7]. Despite significant medical advances, the rate of prematurity has not declined over the past 40 years; rather, it has continued to rise. An increase in maternal age and underlying maternal health problems, such as diabetes and hypertensive disorders or iatrogenic factors like greater use of infertility treatments, which lead to increased rates of multiple pregnancies and changes in obstetric practices (such as more caesarean births before term) [8] have been identified as different reasons that explain this phenomenon. The prevalence of preterm births is about 12 to 13 % in the United States of America and 5 to 9 % in the European Union and other developed countries [5]. Although all deliveries before 37 weeks of gestation are considered preterm, the highest proportion of complications and neonatal death occur in those born at less than 34 weeks.

In order to reach an effective decrease of prematurity, two premises are necessary: to identify pregnant women who are at risk and to dispose of measures that serve to extend pregnancy, thus avoiding the prematurity. Some published papers describe sonographic measurement of the cervix at the 16th week as a method of screening to detect women at risk of spontaneous preterm delivery [3, 9–11]. In 1996 [1], Iams et al. demonstrated that the risk of preterm delivery is inversely proportional to the length of the cervix on transvaginal ultrasonography (US) between 24 and 28 weeks of gestation in a nonselected population in the United States of America.

After this publication [1], several studies have shown that the risk of preterm birth was inversely correlated to the length of the cervix as measured by transvaginal ultrasound [2, 3, 9, 12, 13], which has been confirmed in a recent meta-analysis [14].

Therefore, considering that the risk of prematurity is inversely proportional to cervical length, cervical measurement constitutes a method of screening for use in pregnant women who are asymptomatic before 24 weeks [15–17]. For decades, different types of therapeutic intervention have been explored in order to decrease preterm births, including primary prevention measures (all pregnant women), secondary prevention measures (only in women at risk) or tertiary prevention measures (initiated after the delivery process has started) [18]. A recent review concluded that there was insufficient evidence to provide sound recommendations for clinical practice in approximately half of the evaluated interventions [19]. Nevertheless, according to several trials and systematic reviews, two strategies, vaginal progesterone [20–28] and silicon cervical pessary [29–32], have been shown to be effective at decreasing the rate of spontaneous delivery before 34 weeks of gestation in at-risk women who have a short cervix.

Progesterone is a key hormone during gestation that has uterus-relaxing effects. Low levels of progesterone are associated with a high grade of cervix maturation and, consequently, a shorter cervical length and a higher risk of spontaneous delivery. The administration of progesterone supplements decreases preterm delivery in women with a short cervix [20–23] and in pregnant women with a history of preterm births [25, 26].

In March 2012, Romero et al. published a meta-analysis [27], which explored whether the use of vaginal progesterone in asymptomatic women whose ultrasound showed a short cervix (under 25 mm) in the second trimester reduced the risk of preterm birth and improved neonatal morbidity and mortality.

Five relevant clinical trials, which included a total of 775 women and 827 infants, were analyzed. The results showed that treatment with vaginal progesterone was associated with a significant reduction in the preterm birth rate before 33 weeks (12.4 % versus 22.0 %; relative risk (RR) 0.58; 95 % confidence interval (CI), 0.42 to 0.80), <35 weeks (RR, 0.69; 95 % CI, 0.55 to 0.88), and <28 weeks (RR, 0.50; 95 % CI, 0.30 to 0.81); respiratory distress syndrome (RR, 0.48; 95% CI, 0.30 to 0.76); composite neonatal morbidity and mortality (RR, 0.57; 95 % CI, 0.40 to 0.81); birth weight <1500 g (RR, 0.55; 95 % CI, 0.38 to 0.80); admission to neonatal intensive care unit (RR, 0.75; 95 % CI, 0.59 to 0.94); and requirement for mechanical ventilation (RR, 0.66; 95 % CI, 0.44 to 0.98). There were no significant differences in the rate of adverse maternal events or congenital anomalies between the vaginal progesterone and placebo groups. The authors concluded that the data provides sufficient evidence about the benefit of vaginal progesterone to prevent the preterm birth in women with a sonographic short cervix. They recommend measuring cervical length using transvaginal sonogram at 19 to 24 weeks of gestation.

Although cervical pessaries were introduced for management of cervical incompetence [33–35] at the end of the 1950s, a very limited number of papers regarding the use of pessaries in pregnant women have been found. In a case control study, Arabin et al. [29] observed that the prematurity rate significantly decreased in singleton and twin pregnancies in women with a short cervix in comparison with the controls when pessaries were used.

The first randomized study using a pessary (PECEP trial) was published in 2012 [30]. A total of 385 pregnant women with singleton pregnancies and a cervix length of ≤25 mm detected through vaginal ultrasonography at 18 to 22 weeks of gestation were randomized (1:1) to cervical pessary or expectant management. A significant reduction (76 %) in spontaneous deliveries before 34 weeks was found in the pessary group (6.3 % versus 26.8 %; odds ratio 0.18, 95 % CI 0.08 to 0.37; *P* <0.0001). In addition, a decrease in low-weight neonates (<2500 g) was detected (6.3 % versus 29.5 %; odds ratio 0.23, 95 % CI 0.12 to 0.43; *P* <0.0001). After the PECEP publication, it was suggested [31] that more studies are needed in different settings to confirm the results of this single trial.

Finally, regarding safety, it is worth highlighting that natural, micronized vaginal progesterone is a medicine that can be administered either orally or vaginally. There are no described adverse reactions in the product monograph when it is administered vaginally. Some women have reported a slight increase in vaginal discharge [36]. Cervical pessary can produce an increase in vaginal discharge, but without an increase in bacterial vaginosis or corioamnionitis in pregnant women. Although slight discomfort can sometimes occur during pessary placement, most patients note that the discomfort is acceptable [30].

Because of the published data, ultrasound measurement of the cervix is being progressively incorporated into clinical practice and either pessary or progesterone is increasingly being used in women with a short cervix. However, there is a “therapeutic hole” and neither of the two strategies is approved for this indication. In order to clear up any doubts about the potential benefits of both treatments and to establish clinical recommendations, comparative data are necessary. Alfirevic et al. [37] compared three different cohorts using cervical pessary, vaginal progesterone or cerclage for women with a singleton pregnancy and a high risk of preterm birth due to a prior spontaneous preterm birth before 34 weeks and a shortened cervical length as detected by transvaginal ultrasound. Their data suggests that the three treatments are a reasonable option in this population and they concluded that direct randomized comparisons of these strategies are needed.

The main objective of the PESAPRO trial is to compare the efficacy of two treatments, vaginal progesterone and cervical pessary, in terms of decreasing the rate of spontaneous preterm births before 34 weeks of gestation in women with a short cervix, which has been detected by transvaginal ultrasonography.

## Methods/Design

PESAPRO is a noncommercial, national, multicenter, randomized, open-label trial designed to compare the effect on premature birth prevention of two currently accepted strategies: administration of progesterone (vaginally) or the placement of vaginal pessaries in pregnant women with a short cervix (≤25 mm) as diagnosed by transvaginal ultrasonography (US) in the second trimester morphology ultrasound.

The inclusion of a third arm with placebo control was discussed in the original design because it would have been a valuable tool from a methodological point of view.- However, it was later discarded due to ethical and feasibility issues after taking into consideration the most recent publications.

### Participating centers

Thirty-one hospitals are currently participating in the PESAPRO trial. Fourteen center had initially been invited to participate, and, later, 17 additional hospitals were incorporated into the trial in a second phase.

All participating Obstetrics Units routinely perform transvaginal ultrasonography in the second term as a screening diagnosis method to detect women at risk of preterm birth (listed in Appendix 1).

### Study population and trial development

Pregnant women with a short cervix (≤25 mm) as identified by routine transvaginal ultrasonography at 19 to 22 weeks of gestation are eligible to enroll. Before the evaluation, the woman should have an empty bladder and be placed in the dorsal lithotomy position. The vaginal probe should be placed in the anterior fornix without pressure. Initial orientation is established by locating the sagittal view of the cervix. The length of the endocervical canal should be measured in a straight line from the internal to the external cervix. The cervix is dynamic; therefore, three measurements should be made over a 3-minute period, and the shortest measurement reported for clinical use [16, 38]. During this routine ultrasonography in the second term, singleton-pregnancy women with a short cervix (≤25 mm) are considered to participate in the PESAPRO trial. Table 1 shows the inclusion and exclusion criteria. A member of the research team informs the patient about the clinical trial during this (second trimester ultrasound) visit or at a different time, according to investigator criteria. Once informed consent has been obtained and baseline data has been collected (demographic, medical and obstetric history, vaginal and endocervix samples for culture), participants are individually randomized to vaginal progesterone or pessary. Study participants are evaluated every four weeks (±7 days) for a total of four visits; routine assessments (such as blood pressure, and weight), cervicometry and fetal ultrasonography are performed during each visit to assess adverse events and compliance with the study medication (in the progesterone group). Women are asked to return unused study medication from the previous 4 weeks and compliance is determined on the basis of the amount of the study drug that is unused. Study treatments continue until the third term (37 to 37 + 4 weeks), when the obstetrician removes the pessary, collects all the leftover medication, or until delivery, whichever occurs first.

**Table 1:** Inclusion and exclusion criteria for PESAPRO

Inclusion criteria	Exclusion criteria
• Women older than 18 years old	• Major fetal and/ or uterine abnormalities
• Pregnant women with a short cervix (<25 mm) identified by the use of routine transvaginal ultrasonography at 19 to 22 weeks of gestation	• Placenta previa during current pregnancy
• Gestational age at randomization between 20 weeks + 1 and 23 weeks +6	• Vaginal bleeding or ruptured membranes in the moment of randomization
• Single pregnancy	• Cervical cerclage *in situ*
• Women sign informed consent according to the GCP and local legislation	• History of cone biopsy
	• History of three or more premature labor
	• Allergic to peanut
	• Contraindication for progesterone usage
	• Active treatment with progesterone at randomization
	• If in an investigator’s opinion, there are findings on physical examination, abnormalities in the results of clinical analyzes or other medical factors, social or psychosocial that could negatively influence
	• Women unable to give the informed consent

Any patients who develop preterm labor during the study are treated according to the standard practice of the participating institution. The study treatments continue during hospitalization until the moment at which delivery is unavoidable or until completion of gestation is indicated. One additional assessment (medical record review or phone call) is performed 4 to 6 weeks after delivery in order to collect full data. See Table 2 for the detailed schedule of enrollment and assessments.

**Table 2:** Schedule of PESAPRO study’s visit

	Visit 1 (Routine control 2 term)^a^	Inform Visit	Visit 0 basal (20 weeks + 1 to 23 weeks + 6)	Visit 1 (V0 + 4 weeks)	Visit 2 (V1 + 4 weeks)	Visit 3 (V2 + 4 weeks)	Visit 4 (EOT) (37 weeks to 37 weeks + 4)	Post-delivery visit (4 to 6 weeks post-delivery)
Eligibility criteria		**√**	**√**					
Informed consent			**√**					
Randomization and start of treatment			**√**					
Blood pressure/ weight			**√**	**√**	**√**	**√**	**√**	
Vaginal culture			**√**					
Manual vaginal exam			**√**				**√**	
Cervicometry	**√**		**√**	**√**	**√**	**√**	**√**	
Follow-up Questionnaire				**√**	**√**	**√**	**√**	
Basic fetal US			**√**	**√**		**√**	**√**	
End of treatment (EOT)							**√**	
Review maternal medical history			**√**					**√**
Review neonate medical history								**√**

^a^This visit is a routine (“standard of care”) visit in the control of pregnancy. Patients with a short cervix are invited to an extra visit (for information) before being enrolled in the study

### Randomization

Consecutive eligible patients are randomly allocated in a 1:1 ratio to one of the two treatment groups: vaginal progesterone or cervical pessary. The randomization sequence is computer generated (by EpiDat program). It is protected and it is managed exclusively by the Clinical Pharmacology Unit at University Hospital Puerta de Hierro - Majadahonda, which has no role in recruitment. The investigators receive the patient’s identification (ID) number and the assigned treatment by a central phone call, after having been informed that a new patient has entered into the trial. An auditable registry of the date and time of inclusion, patient ID number and treatment assignment is stored in the sponsor’s records.

### Study interventions

Two accepted treatments in the management of preterm birth risk are being assessed in the PESAPRO trial, although neither of them is approved for use in this condition. For statistical purposes, we consider the pessaries to be the experimental group and progesterone to be the reference (or comparator) group. Patients who are assigned to the comparator group receive 200 mg of micronized progesterone per day by vaginal route; women self-administer the medicine once daily, preferably before going to bed.

The cervical pessary used in this study is a perforated-cerclage type pessary, a hypoallergenic silicon medical device certified by European Conformity, size 65/25/32 (65 mm lower larger diameter, 25 mm height and 32 mm upper smaller diameter). The pessary is disposed of (and removed) by qualified personnel (Obstetrician) at the clinic. Both treatments are withdrawn between the 37th week and 37 week + 4.

### Treatment registry

During the trial, both treatments are being properly registered. The commercial progesterone used (PROGGEFFIK™) is re-labelled in the Clinical Trials Pharmacy Unit of the University Hospital Puerta de Hierro - Majadahonda and then distributed to all participating centers. The label includes the following data: trial code, sponsor name, investigational product, batch number, expiration date and the trial subject identification number. Regarding the pessary, the batch number and the expiration date also are being registered.

### Study outcomes

The primary outcome measure is the proportion of spontaneous preterm birth before 34 weeks of gestation. The secondary outcomes are the following: proportion of spontaneous preterm birth before the 37th and 28th weeks, the rate of premature rupture of membranes before 34 weeks, the neonate’s weight at birth, the rate of fetal intrauterine mortality during the treatment period, the rate of neonatal morbidity and mortality, the need for hospitalization and tocolysis treatment, the rate of chorioamnionitis during the third term, symptomatic vaginal infections during the treatment period and the proportion of participants with adverse events.

### Statistical analysis

A sample size of 254 women (127 women per arm) was predefined to show the noninferiority of the experimental group (pessary) versus the reference group (progesterone), assuming a proportion of preterm birth (before 34 weeks) of 6 % and 12.4 %, respectively, with a noninferiority margin of 4 %, a 2.5 % one-sided alpha level, a statistical power of 80 % and a drop-out rate of 5 %.

Statistical analysis will be based on the intention-to-treat principle and a sensitivity analysis using the per-protocol subset for the main outcome will be used for sensitivity purposes. The noninferiority hypothesis will be tested by estimating treatment rate differences against the noninferiority margin. The survival function and the median (95 % confidence interval (CI)) time to delivery will be estimated by means of the Kaplan-Meier method and treatment effects will be compared using the log-rank test and hazard ratios (HR) (95% CI) will be taken from the Cox model. The rest of the variables will be analyzed using the Fisher’s exact test to compare categorical data, the t-test for continuous variables and the Mann- Whitney test for ordinal and non-normally distributed variables.

The analysis will be performed using SAS v9.2, or a newer version, software (SAS Institute Inc., Cary, NC, USA), and the level of significance will be set at 5 % (two-sided).

### Organization of the trial

PESAPRO is a noncommercial trial organized by the Obstetrics and Gynecology Department and the Clinical Pharmacology Unit of the University Hospital Puerta de Hierro - Majadahonda. The sponsor role is taken by C. Martinez-Payo. The medical coordinator of the trial is S. Cruz Melguizo. The Clinical Pharmacology Unit oversees the methodological and organizational aspects of the study. Since 2014, the study has been receiving support from the Spanish Clinical Research network (SCReN) for regulatory and administrative support, monitoring, data management and pharmacovigilance. The PESAPRO study group comprised of all clinical investigators at the participating centers and the collaborators from the Clinical Pharmacology Unit.

#### Funding/Support

The study is fully funded with public funds obtained in competitive calls: grant PT13/0002/0005 (SCReN-Spanish Clinical Research Network) from the National R+D+I 2013-2016 Plan of the Institute of Health Carlos III (AES 2013), grant EC11/086 of the Ministry of Health Call for Independent Clinical Research in year 2011, and grant PI12/02240 from the Institute of Health Carlos III, ISCIII, from the National I+D+I 2008-2011 Plan and co-financed by FEDER funds.

#### Coordination and conduct of the trial

The study is coordinated by the Obstetrics and Gynecology Unit and the Clinical Pharmacology Unit (Appendix 2). Three investigator meetings have been held in order to review the study protocol and data collection in the electronic case report form (eCRF). Additionally, after the necessary approvals were obtained, a meeting was held in each hospital before the start of patient recruitment procedures. All documents required for the study are available at each participating site. The eCRF is a secure, interactive, web-response system available at each study center, provided and managed by the Biostatistics and Data Management Core Facility of Institut d’investigacions Biomèdiques August Pi i Sunyer (IDIBAPS) - Hospital Clínic, Barcelona.

In each participating Obstetrics Unit, the physicians and research personnel (research nurse or assistant) are in charge of patient screening and inclusion, ensuring compliance with the study protocol and collecting the study data in the eCRFs. The trial is being monitored by an independent monitor. The monitor regularly reviews the eCRFs, ensuring compliance with the trial protocol, data completeness and data accuracy. All adverse events and serious adverse events are being recorded according to standard procedures.

### Ethical considerations

The trial has been registered at European Clinical Trial Database (EUDRACT2012-000241-13) and at ClinicalTrials.gov (NCT01643980). The study is being conducted in accordance with Good Clinical Practice guidelines, the guiding principles of the Declaration of Helsinki, and applicable local regulations. The study was approved by the Spanish National Competent Authority (AEMPS) and by the Research Ethics Committees of the participating centers (listed in Appendix 3). Any relevant protocol amendments will be submitted to the competent Ethics Committee for review and will be updated in the trial registries. Participants are provided with information sheets and written informed consent is obtained prior to recruitment and baseline assessment.

A specific insurance policy has been contracted to cover compensations to patients in the event of injuries, in compliance with the requirements of Spanish law regarding clinical trials.

Although a third control arm with placebo is very valuable from a methodological point of view in a noninferiority comparative trial, it was discarded in our trial. The main reason was related to the fact that most participant hospitals were already treating these women with a short cervix (either with a pessary or with progesterone) as a measure to decrease the risk of preterm birth, based on recent publications. Although confirming data with regards to the superiority of both treatments versus placebo would have been highly valuable, it was considered ethically questionable.

## Discussion

Preterm birth is a major public health problem with a relevant neonatal morbimortality. It has been established that a short cervix (≤25 mm) diagnosed by transvaginal ultrasonography in the second trimester constitutes an important risk factor of preterm delivery [1, 9, 11, 39]. Although several therapeutic strategies (vaginal progesterone, cervical pessary and cerclage) have been used in these women showing a potential benefit, they have never been compared to one another in randomized trials. This study will assess the comparative efficacy of two of these three treatments (vaginal progesterone versus pessary). It is possible that the results of this study will contribute to the establishment of clinical recommendations regarding the use of both treatments in the prevention of preterm delivery in women with a short cervix.

## Trial status

Patient inclusion to the PESAPRO trial began in September 2012. Enrollment is ongoing. The study is being conducted at 29 investigational sites. As of May 2015, 190 patients had been included in the study. Patient recruitment is expected to end in July 2015.

## Appendix 1

**Table 3:** PESAPRO trial sites and principal investigators

Site ID	Trial sites	Principal investigators
1	Hospital Puerta de Hierro Majadahonda	Dra. Cristina Martínez Payo
		Dra. Sara Cruz Melguizo
2	Hospital Príncipe de Asturias	Dra. Estefanía Cordero
3	Hospital de Alcorcón	Dr. Begoña Adiego Burgos
4	Hospital General Yagüe	Dr. Javier Martínez Guisasola
5	Hospital de Getafe	Dr. Luis Martínez Cortés
6	Hospital de León	Dr. Celso García González
7	Hospital Río Hortega	Dr. Gonzalo Quesada Segura
8	Hospital Ramón y Cajal	Dr. Leopoldo Abarca
9	Hospital Miguel Servet	Dr. Ricardo Savirón
11	Hospital Infanta Elena	Dra. Esther Pérez Carbajo
12	Hospital InfantaSofía	Dr. José Alberto Rodríguez León
13	Hospital La Paz	Dr. José Luis Bartha
14	Hospital de Fuenlabrada	Dra. MaríaTeulón
16	Hospital Universitario Rey Juan Carlos I	Dra. Rosa Nogales
17	Hospital UniversitarioQuiron Dexeus	Dr. Rodríguez
18	Hospital UniversitarioSevero Ochoa	Dra. Gregoria Alonso
19	Hospital Clínico Universitario de Valladolid	Dra. Cristina Álvarez Colomo
20	Hospital General Universitario de Ciudad Real	Dra. M Ángeles Anaya Baz
21	Hospital Quirón Málaga	Dr. Daniel Abehsera Davó
22	Hospital Universitario de Guadalajara	Dra. Maria Jesús Cancelo
23	Hospital Sanitas la Zarzuela	Dra. Elisa Maria Díaz De Teran
24	Hospital d´Igualada	Dr. Joan Carles Mateu Pruñunosa
25	Hospital Madrid UniversitarioMonteprincipe	Dra. Gloria Gálvez
26	Hospital Universitario Reina Sofía	Dr. Antonio de la Torre
27	Hospital Palamos (ComarcalBaixEmporda)	Dr. José Manuel Marqueta Sánchez
28	Hospital Universitario San Juan de Alicante	Dra. Rosa Bermejo
29	Hospital Universitario de Donostia	Dra. Arantza Lekuona Artola
30	Hospital Sanitas La Moraleja	Dr. Eduardo Cabrillo Rodríguez
31	Hospital Universitario de Móstoles	Dra. Emilia de Dios

## Appendix 2

### Steering committee

Sara Cruz Melguizo, Cristina Martínez-Payo, Luis San Frutos, Francisco López Sánchez, Cristina Avendaño Solá, Belén Ruiz Antorán, and Lourdes Cabrera García.

### Data safety monitoring committee

- Dra. Caridad Pontes: Consultant Physician at Hospital de Sabadell.
- Dr. José Ríos: Statistician Coordinator of the Platform of Biostatistics and data Management core and facilityb at the IDIBAPS (Hospital Clinic Barcelona).
- Dr. Tirso Pérez: Head of Gynecology at Hospital Universitario Puerta de Hierro Majadahonda.

## Appendix 3

**Table 4:** Research Ethics Committees that approved the study in the various centers involved

Site ID	Trial sites	Principal investigators	Research Ethics Committees ethical
1	Hospital Puerta de Hierro-Majadahonda	Dra. Sara Cruz Melguizo	CEIC H.U. Puerta de Hierro-Majadahonda (REFERENCE)
2	Hospital Príncipe de Asturias (Alcalá de Henares)	Dra. Estefanía Cordero	CEIC H.U. Príncipe de Asturias
3	Hospital de Alcorcón	Dr. Begoña Adiego Burgos	CEIC Fundación Hospital Alcorcón
4	Hospital General Yagüe (Burgos)	Dr. Javier Martínez Guisasola	CEIC Área de Salud de Burgos y Soria
5	Hospital de Getafe	Dr. Luis Martínez Cortés	CEIC H.U. Getafe
6	Hospital de León	Dr. Celso García González	Área de Salud de León
7	Hospital Río Hortega (Valladolid)	Dr. Gonzalo Quesada Segura	Área de Salud de Valladolid Oeste
8	Hospital Ramón y Cajal	Dr. Leopoldo Abarca	CEIC H.U. Ramón y Cajal
9	Hospital Miguel Servet (Zaragoza)	Dr. Ricardo Savirón	Aragón - CEICA
10	Hospital Marqués de Valdecilla	Dr. Gerardo Ballesteros	CEIC de Cantabria
11	Hospital Infanta Elena (Valdemoro)	Dra. Esther Pérez Carbajo	CEIC -FJD
12	Hospital Infanta Sofía	Dr. José Alberto Rodríguez León	CEIC H.U. La Paz
13	Hospital La Paz	Dr. José Luis Bartha	CEIC H.U. La Paz
14	Hospital de Fuenlabrada	Dra. María Teulón	CEIC H.U. Fuenlabrada
15	Hospital La Fe (Valencia)	Dr. Vicente José Diago Almela	CEIC H.U. La Fe
16	Hospital Universitario Rey Juan Carlos I	Dra. Rosa Nogales	CEIC -FJD
17	Hospital Universitario Quiron Dexeus	Dr. Rodríguez	CEIC H. U. Quirón Dexeus
18	Hospital Universitario Severo Ochoa	Dra. Gregoria Alonso	CEIC H. U. Severo Ochoa
19	Hospital Clínico Universitario de Valladolid	Dra. Cristina Álvarez Colomo	CEIC Área de Salud de Valladolid Este
20	Hospital General Universitario de Ciudad Real	Dra. M Ángeles Anaya Baz	CEIC H. Gral. U. de Ciudad Real
21	Hospital Quirón Málaga	Dr. Daniel Abehsera Davó	CEIC Andalucía
22	Hospital Universitario de Guadalajara	Dra. Maria Jesús Cancelo	CEIC H. Gral. U. de Guadalajara
23	Hospital Sanitas la Zarzuela	Dra. Elisa Maria Díaz De Teran	CEIC H.U. Puerta de Hierro-Majadahonda
24	Hospital d´Igualada	Dr. Joan Carles Mateu Pruñunosa	CEIC del Hospital Universitari Bellvitge
25	Hospital Madrid Universitario Monteprincipe	Dra. Gloria Gálvez	CEIC Hospital de Madrid
26	Hospital Universitario Reina Sofía	Dr. Antonio de la Torre	CEIC Andalucía
27	Hospital Palamos (Comarcal Baix Emporda)	Dr. José Manuel Marqueta Sánchez	CEIC Institut d.Assistencia Sanitaria de Girona
28	Hospital Universitario San Juan de Alicante	Dra. Rosa Bermejo	CEIC Hospital General Universitario San Juan de Alicante
29	Hospital Universitario de Donostia	Dra. Arantza Lekuona Artola	CEIC de Euskadi
30	Hospital Sanitas La Moraleja	Dr. Eduardo Cabrillo Rodríguez	CEIC H.U. La Paz
31	Hospital Universitario de Móstoles	Dra. Emilia de Dios	CEIC H Universitario de Móstoles

## Abbreviations

CI: confidence interval
CMH: Cochran-Mantel-Haenszel
eCRF: electronic case report form
IDIBAPS: Institutd’investigacions Biomèdiques August Pi i Sunyer
ITT: intention to treat
ISCIII: Institute of Health Carlos III
PP: per protocol
RCT: randomized clinical trials
RR: relative risk
SCReN: Spanish Clinical Research Network
US: ultrasonography
USA: United States of America
